# The Impact of Panic Disorder on Severe Asthma and Chronic Rhinosinusitis with Nasal Polyps: A Comparative Clinical Profile Study

**DOI:** 10.1192/j.eurpsy.2025.1017

**Published:** 2025-08-26

**Authors:** D. Caldirola, S. Daccò, F. Puggioni, M. Crebelli, M. Grassi, A. Alciati, L. Malvezzi, E. Heffler, G. Perna

**Affiliations:** 1 Department of Biomedical Sciences, Humanitas University, Pieve Emanuele, 20090 Milan; 2 IRCCS Humanitas Research Hospital, Personalized Medicine, Asthma, and Allergy, Rozzano 20089, Milan, Italy; 3 IRCCS Humanitas Research Hospital, Rozzano, 20089 Milan, Italy; 4 Department of Otorhinolaryngology and Head and Neck Surgery, IRCCS Humanitas Research Hospital, Rozzano, 20089 Milan, Italy

## Abstract

**Introduction:**

Severe asthma (SA), often associated with chronic rhinosinusitis with nasal polyps (CRSwNP), contributes significantly to global disability and mortality. A personalized treatment approach, including addressing treatable traits like mental health, is crucial for improving outcomes. The impact of panic disorder (PD) on asthma symptoms and outcomes requires investigation due to their epidemiological association.

**Objectives:**

To cross-sectionally compare the clinical presentation of outpatients with SA and/or CRSwNP, with and without PD, treated at the Personalized Medicine Asthma and Allergy Clinic, Humanitas Research Hospital.

**Methods:**

Participants were consecutively recruited among outpatients attending follow-up visits to treat SA and/or CRSwNP from February to March 2024. All were previously enrolled in the SANI (Severe Asthma Network Italy) or RINET (Rhinosinusitis Italian Network) registries. Participants completed a comprehensive self-report survey on sociodemographic, lifestyle, medical, and psychiatric information, along with validated questionnaires assessing asthma control, severity of nasal obstruction, burden and emotional responses to physical symptoms, psychophysical well-being, and the PD module from the Patient Health Questionnaire (PHQ) screening tool, to identify provisional diagnoses for current or past PD. Data were analyzed using the Kruskal-Wallis, post-hoc Dunn’s, and Fisher’s exact tests, with Holm’s adjustment for multiple comparisons. The significance level was set at 0.05.

**Results:**

Seventy-nine patients, 46 women (58.2%) and 33 men (41.8%), participated in this study. Thirty patients (38%) had SA only, 44 (55.7%) had both SA and CRSwNP, and 5 (6.3%) CRSwNP only. Current PD was identified in 7 outpatients (8.9%), while 12 (15.2%) had past PD. Compared to patients who have never experienced PD, those with current PD had significantly worse asthma control, more severe nasal obstruction, greater dyspnea and physical symptom burden, as well as greater proneness to catastrophizing about asthma, heightened attentional focus on internal bodily sensations, and lower quality of life. Patients with past PD had greater dyspnea and physical symptom burden, swallowing difficulty, and reduced quality of life compared to those without PD. No significant differences were found between current and past PD groups.

**Conclusions:**

The prevalence of current or past PD among patients with SA and/or CRSwNP was approximately three times higher than in the general population, corroborating previous epidemiological findings. PD was associated with poorer asthma and nasal symptom control, along with a higher burden and sensitivity to respiratory and physical symptoms. Our preliminary results suggest a need for PD screening and targeted interventions for these patients. Further studies with psychiatric interviews and objective respiratory measures are warranted.

**Disclosure of Interest:**

None Declared

